# Modifications of Own Mothers’ Milk Fortification Protocol Affect Early Plasma IGF-I and Ghrelin Levels in Preterm Infants. A Randomized Clinical Trial

**DOI:** 10.3390/nu11123056

**Published:** 2019-12-14

**Authors:** Eleni Agakidou, Thomais Karagiozoglou-Lampoudi, Elisavet Parlapani, Dimitrios J. Fletouris, Kosmas Sarafidis, Vasiliki Tzimouli, Elisavet Diamanti, Charalampos Agakidis

**Affiliations:** 1 1st Department of Neonatology & NICU, Aristotle University of Thessaloniki, Ippokration General Hospital, Konstantinoupoleos 49, 54246 Thessaloniki, Greece; parlapanielli@hotmail.com (E.P.); kosmas.sarafidis@gmail.com (K.S.); diamanti@med.auth.gr (E.D.); 2 Department of Nutrition and Dietetics, Alexander Technological Educational Institute of Thessaloniki, 57400 Thessaloniki, Greece; thomaiskl@gmail.com; 3 Laboratory of Milk Hygiene and Technology, School of Veterinary Medicine, Faculty of Health Sciences, Aristotle University of Thessaloniki, 54124 Thessaloniki, Greece; djflet@vet.auth.gr; 4 1st Pediatric Department, Aristotle University of Thessaloniki, Ippokration General Hospital, Konstantinoupoleos 49, 54246 Thessaloniki, Greece; vtzimouli@gmail.com (V.T.); cagakidis@gmail.com (C.A.)

**Keywords:** BMI, ex-preterm children, feeding preterm infants, growth, human milk fortification, nutrition, programming, protein intake

## Abstract

The aim was to investigate the effect of two own mother’s milk (OMM) fortification protocols on (a) IGF-I and ghrelin plasma levels at 35 post-conceptional weeks (PCW, T2) and whether this effect is maintained after elimination of the differences in OMM fortification, and (b) growth until 12 months corrected age. Forty-eight OMM-fed preterm infants (GA 24–32 weeks) were randomly allocated to the fixed-fortification (FF) group (*n* = 23) and the protein-targeting fortification (PTF) group (*n* = 25) targeting the recommended daily protein intake (PI). Plasma IGF-I and ghrelin were assessed at 35 (T2) and 40 (T3) PCW while growth was longitudinally assessed until 12 months corrected age. PTF group had lower IGF-I and higher ghrelin than FF group at T2, while receiving lower daily protein and energy amounts. PI correlated positively to T2-IGF-I and inversely to T3-ghrelin while energy intake (EI) correlated inversely to T2- and T3-ghrelin. Group and PI were independent predictors of adjusted T2-IGF-I, while group and EI were predictors of adjusted and T2-ghrelin. Growth parameter *z*-scores were comparable between groups up to 12 months corrected age. Modifications of OMM fortification have a transient effect on early plasma IGF-I and ghrelin levels in preterm infants in a way consistent with the previously recognized protein-energy/endocrine balance, indicating a potential programming effect.

## 1. Introduction

Feeding preterm infants own mother’s milk (OMM) has major advantages for its health, including reduced neonatal mortality and morbidity, better neurodevelopment, and potentially lower risk of developing metabolic syndrome in adulthood [1,2,3]. However, preterm infants have increased macro- and micro-nutrient requirements, which are difficult to be met by OMM or donor human milk [4,5]. Therefore, fortification of OMM with proteins and other macro- and micro-nutrients is recommended in order to support the infant’s growth [6,7]. So far, three methods of human milk fortification have been used; the standard or fixed fortification (FF), in which a fixed amount of fortifier is added to OMM or donor milk; the individualized adjustable fortification, which takes into consideration the blood urea levels; and the individualized targeted fortification, based on human milk analysis and targeting the recommended daily macronutrient intake [6,7]. In clinical practice, the multicomponent human milk fortifier (HMF) formulations are those used primarily, whereas single component formulations are often unavailable and their use may be more time-consuming.

The rapid growth and development during the fetal and early postnatal phases render them the most critical periods of metabolic programming [8]. During these vulnerable stages of life, nutrition presents as a major environmental factor that can promote programming of the metabolic and endocrine mechanisms regulating the energy balance [9,10]. However, the role of early nutrition on later development of metabolic syndrome has not been fully elucidated [3]. Recent research focuses on the potential association of early nutrition modifications with metabolism-related hormones, including insulin-like growth factor—I (IGF-I) and ghrelin, which play an important role in growth and development during both fetal and early postnatal life, as well as in energy regulation [11,12].

It has been reported that high protein intake by full-term infants either stimulates [13,14] or has no effect [15] on IGF-I production. In preterm infants, nutrition and insulin are critical for IGF-I production, which is also affected by genetic factors [12]. Clinical studies relate IGF-I with both growth and nutritional status of preterm infants [16,17]. Regarding circulating ghrelin in neonates, existing data focuses on the association of cord blood and plasma levels during the first eight postnatal weeks with intrauterine and early postnatal growth reporting conflicting results [15,18,19,20,21,22,23,24]. Moreover, ghrelin levels at pre-pubertal age have been found higher in ex-preterm children than in the full-term ones [19]. So far, there is no published data regarding the effect of human milk fortification protocols on early IGF-I and ghrelin levels of preterm infants.

Studies on the “early protein” hypothesis have suggested the first 2–4 months of life as a “window opportunity” for metabolic programming. For this reason, we designed this prospective study at the most vulnerable to programming period of preterm infants’ extrauterine life, i.e., from birth to term equivalent age. In the current study, we implemented two OMM fortification protocols; a modified, partly individualized OMM fortification protocol adjusted to the measured OMM protein content and targeting the recommended protein intake and a FF protocol, by using a multicomponent HMF. The primary aims of this study were to compare the effect of the modified, protein-targeting fortification (PTF) protocol vs. the FF one (a) on IGF-I and ghrelin plasma levels up to the 35th week of postconceptional age (PCA), and whether this effect is maintained after elimination of the differences in fortification regimens, and (b) on growth up to 12 months of corrected age (CA), compared to the FF protocol. The secondary aim was to examine the effectiveness of the two OMM fortification protocols in attaining the recommended range of macronutrient intake.

## 2. Materials and Methods

### 2.1. Study Design and Population

This is a single-center, randomized, double-blind study with parallel design (two treatment groups), and allocation ratio 1:1. Preterm neonates with a gestational age 25–32 weeks and birth weight less than 1500 g, admitted within the first 24 h of life to a tertiary neonatal intensive care unit between March 2013 and March 2016 were assessed for eligibility. Eligible for enrollment were all appropriate-for-gestational-age preterm infants whose mothers intended to provide them with their own breast milk.

### 2.2. Exclusion Criteria

Exclusion criteria included evidence of maternal health problems precluding breastfeeding, congenital infections, metabolic/genetic syndromes, early death, intra-peri-ventricular hemorrhage of grade III-IV, sepsis and/or necrotizing enterocolitis, and consent refusal. Post-randomization exclusion criteria included death prior to the 40th week of PCA, interruption of enteral or exclusive OMM feeding for more than 3 days for various reasons (i.e., inadequate OMM supply, feeding intolerance, sepsis and/or necrotizing enterocolitis), moderate/severe bronchopulmonary dysplasia, and withdrawal of parental consent.

### 2.3. Ethics

All parents gave their written informed consent for inclusion before joining in the study. The study was conducted in accordance with the Declaration of Helsinki, and the protocol was approved by the Ethics Committee of the Faculty of Medicine of Aristotle University of Thessaloniki and the Scientific Committee of the Hospital. The study was registered with the US National Institute of Health at www.ClinicalTrials.gov (number: NCT01947972).

### 2.4. Intervention and Randomization

Immediately after birth (time-point 0, T0) all study infants were commenced on total parenteral nutrition according to the standard protocol of the neonatal unit. Minimal feedings with OMM was initiated as soon as maternal colostrum was available. In both groups, fortification started as soon as enteral nutrition reached 100 mL·kg^−1^·d^−1^ (T1). All infants in both intervention groups were fed exclusively OMM fortified with a cow’s milk-based, multi-nutrient HMF (PreNAN FM 85, Nestlé) containing 0.20 g of protein, 0.66 g of carbohydrates, 0.004 g of fat, and 3.48 kcal per 1 g of fortifier.

During the week preceding OMM fortification initiation, eligible neonates were randomly allocated into two groups; the fixed fortification (FF) group, which received FF with 5 g of HMF per 100 mL of OMM providing 1 g of protein per 100 mL OMM, and the protein-targeting fortification (PTF) group, in which fortification was adjusted to protein content of OMM, birth weight, and daily amount of milk intake in order to attain the recommended daily protein intake (4–4.5 g·kg^−1^ for infants with a birth weight <1200 g and 3.5–4.0 g·kg^−1^ for infants with a birth weight of 1200–1500 g) [6]. Lactose, fat, and energy content of OMM and HMF were not taken into account when calculating the amount of HMF given to the PTF group. Allocation was performed through a computer-generated randomization list. Cluster randomization was performed based on birth weight below and equal/over 1200 g. Only a member of the nursing staff that was not involved in the infants’ care and clinical/ laboratory assessment was aware of the group assignment. The same person was also responsible for the precise measurement of the quantity of HMF and the distribution of the proper portion (divided into eight feeds) for each participant. The daily feeding volume was at the discretion of the attending neonatologist according to the neonatal unit’s protocol and ranged between 140 and 180 mL·kg^−1^·d^−1^, depending on infant’s weekly weight gain.

In the PTF group, adjustment of fortification to OMM protein content and daily volume of milk intake continued until the 35th week PCA (T2) and then fortification was switched to the FF protocol in order to assess the potential of maintaining the differences in hormone levels between the two groups after elimination of the protocol-associated differences in nutrient intake. Thereby, milk fortification continued beyond T2 and up to the 40th week PCA (T3) using the FF regimen in both groups. The period T1 to T2 corresponds to the intervention period and the period T1 to T3 to the study period.

### 2.5. Breast Milk Collection Protocol and Analysis

Soon after delivery, all mothers eligible for enrollment received written and verbal instructions on breast milk collection and storage, using standard procedures. Analysis of OMM was performed in both groups starting on the day prior to T1 and repeated at weekly intervals, on the same day every week, as in previous studies [25,26,27,28]. Milk analysis was performed by applying mid-infrared spectrometry, using the Milkoscan TM Minor (FOSS Analytical A/S, Hillerod, Denmark). The analyzer was calibrated, according to ISO 9622-IDF (International Dairy Federation) 141-2013, against chemical reference methods for the quantification of macronutrient content (i.e., fat, protein, and carbohydrates) in breast milk (see Supplementary Material for details).

### 2.6. Data Collection

Recorded data included family medical history, antenatal, perinatal, and neonatal data, growth assessment, feeding volumes, and signs of feeding intolerance (abdominal distension, bilious or increased gastric residuals, and emesis). Mean daily energy (kcal·kg^−1^·d^−1^), protein (g·kg^−1^·d^−1^), carbohydrate (g·kg^−1^·d^−1^), and lipid (g·kg^−1^·d^−1^) intake were calculated including milk content and the amount of HMF as well as the parenterally administered macronutrients. Lactose intake was calculated from the total carbohydrates measured in OMM assuming a 20% of total carbohydrates as non-metabolized oligosaccharides [29,30,31,32,33,34,35]. Energy intake was calculated from the macronutrient concentrations by using the Atwater factors: 9 kcal/g for fat and 4 kcal/g for protein and estimated lactose.

### 2.7. Growth Assessment

The weight was measured daily whereas head circumference (HC) and length were measured weekly up to the 40th week PCA using standard procedures. Specifically, nude weight was determined using an electronic balance with an accuracy of ±5 g. Crown-heal length was measured in the supine position to the nearest 0.1 cm using a Holtain neonatometer. Occipitofrontal head circumference was determined as the largest measurement taken across the occiput and forehead using a paper tape. Early growth data was recorded at birth (T0), T1, T2, and T3. Growth was followed up until the 12th month CA as part of routine protocol, and growth data at 3, 6, and 12 months of CA (T4, T5, and T6, respectively) was recorded. Growth parameters were transformed into z-scores using the Fenton 2013 research bulk calculator (available at https://www.ucalgary.ca/fenton/2013 chart) up to the 40th week PCA and then the WHO growth curves and z-scores (available at https://www.who.int/childgrowth/standards/chart_catalogue/en/). Mean changes (Δ) in z-scores of weight, length, HC, and BMI during intervention (T1 to T2) and study periods (T1 to T3) were calculated.

### 2.8. Hormone Assessment

IGF-I and ghrelin levels were assessed at T2 and T3 in plasma samples (1 mL) that were collected 40–60 min before the morning feeding, concomitantly with blood sampling for routine tests, and stored at −80 °C until analysis. IGF-I was measured using IGF-I ELISA (Mediagnost Inc., Reutlingen, Germany) with a sensitivity of 0.09 ng/mL. Total ghrelin was assessed using an Enzyme Immunoassay (Phoenix Pharmaceuticals Inc., Karlsruhe, Germany) with a sensitivity of 130 pg/mL. All measurements were performed twice and the mean values were calculated.

### 2.9. Statistical Analysis

Continuous variables were presented as means and standard deviations while hormone levels as medians and min-max to facilitate comparisons with previous studies. Categorical variables were expressed as counts, proportions, and odds ratios with 95% confidence intervals. Significance of the difference between groups was evaluated using the Mann–Whitney U test, and Fisher’s exact test, for continuous and categorical variables, respectively. Bivariate correlations were performed using the Spearman Correlation Coefficient. Regression analyses were performed using the Generalized Linear Models with dependent variables the natural logarithms of IGF-I and ghrelin levels at T2 and T3, in separate models, and predictors the allocation group, protein, and energy intake. Statistical significance level was set at *p* < 0.05. Analyses were performed with the SPSS version 23 (IBM SPSS Statistics corporation, Chicago, IL, USA).

### 2.10. Sample Size Calculation

Based on preliminary results, we estimated that a sample size of 40 infants (20 in each group) would detect a difference equal to half standard deviation of IGF-I and ghrelin levels at the end of intervention with a power of 80% at 5% two-sided level of significance for both the IGF-I and ghrelin. Assuming a post-randomization drop off of about 40% we estimated that 66 neonates should be enrolled.

## 3. Results

### 3.1. Study Population Characteristics

**Figure 1:**
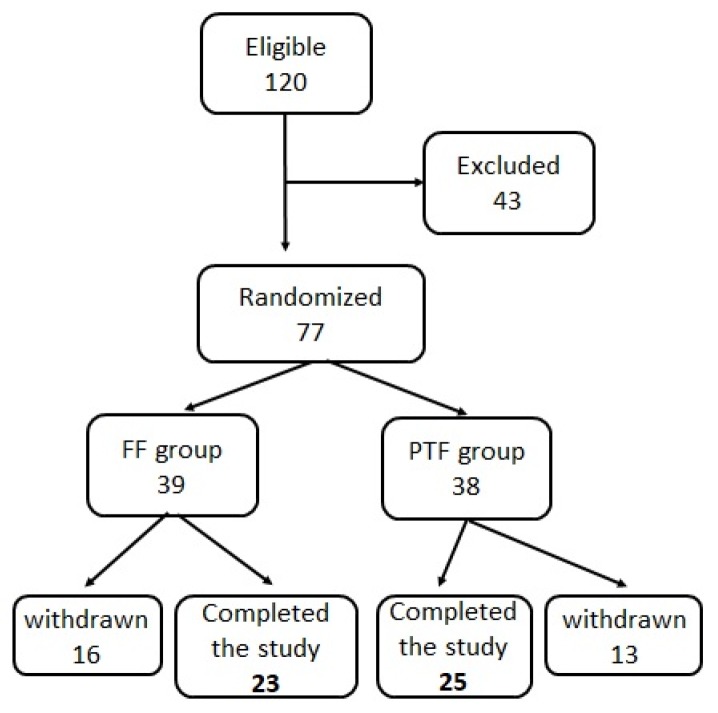
Flow diagram of the study population.

During the study period, 120 infants were eligible for the study, as shown in the cohort flow chart (Figure 1). Forty-three infants met the pre-randomization exclusion criteria (early death in two, inadequate milk supply in 33, suspected congenital infection in one, IVH of grade III/IV in three, sepsis and/or necrotizing enterocolitis in two, family history of allergy in two) and the remaining 77 were randomized, 39 and 38 in the FF and PTF groups, respectively. Twenty-nine infants were excluded following randomization, 16 from the FF group (moderate/severe bronchopulmonary dysplasia in four, sepsis/necrotizing enterocolitis in two, sepsis-related death in one, feeding intolerance in three, inadequate milk supply in six) and 13 from the PTF group (moderate/severe bronchopulmonary dysplasia in 3, sepsis/necrotizing enterocolitis in 1, feeding intolerance in 2, inadequate milk supply in 7). Forty-eight (48) neonates—23 and 25 in the FF and PTF groups, respectively—comprised the study population. The two groups were comparable as regards to the perinatal characteristics and neonatal problems, age at fortification initiation (T1), as well as the duration of intervention and hospitalization (Table 1).

### 3.2. Nutrient Intake and Growth

**Figure 2:**
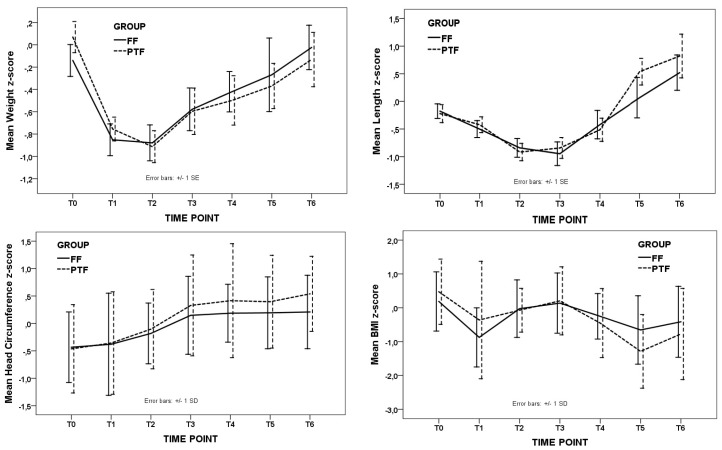
Longitudinal changes in the z-scores of weight, length, head circumference, and BMI, from birth to the 12th month corrected age (CA). FF, fixed fortification; PTF, protein-targeting fortification; T0, birth; T1, start of intervention; T2, end of intervention; T3, 40th postconceptional week; T4, 3 months CA; T5, 6 months CA; T6, 12 months CA. There were no significant differences between the two study groups.

During the intervention period, the two groups received comparable mean daily feeding volumes while the PTF group received significantly lower daily amounts of HMF, and protein, and lower energy of marginal significance compared to the FF group. The estimated amount of lactose intake was significantly lower in the PTF group (*p* < 0.001). After the end of intervention and up to T3, the amount of milk, nutrient, and energy intake did not differ between the two groups. Changes in the z-scores of growth parameters during the intervention period and up to T3 were comparable in the two groups (Table 2). Moreover, z-scores of weight, length, HC, and BMI at each time point up to the 12th month of corrected age did not differ significantly between the two study groups (Figure 2).

### 3.3. Plasma Levels of IGF-1 and Ghrelin in the Two Groups and Correlation with Nutrient Intake and Growth

Compared to the FF group, the PTF group had significantly lower IGF-1 and higher ghrelin levels at T2 but comparable levels at T3 (Table 3). Bivariate correlations of the entire population at T2 showed that the IGF-I positively correlated to the mean daily protein intake while ghrelin inversely correlated to energy intake. At T3, ghrelin negatively correlated to protein and energy intake (Table 4).

Hormone levels at T2 and T3 were not associated with GA and birth weight. IGF-I at T2 positively correlated to Δ z-scores of length during the intervention and up to the 40th week of PCA, while IGF-I at T3 positively correlated to Δ z-scores of weight during intervention and BMI up to the 40th week of PCA. Ghrelin levels at T2 and T3 inversely correlated to the Δ z-score of BMI during intervention (Table 4).

### 3.4. Multivariate Linear Regression

In order to investigate the possible independent effect of group allocation, protein, and energy intake on IGF-I and ghrelin levels at T2 and T3, we constructed separate linear multivariate regression analysis models for each hormone at T2 and T3, as shown in Table 5.

The allocation group and protein intake were significant independent predictors of the adjusted IGF-I at T2. The allocation group and energy were significant independent predictors of the adjusted ghrelin at T2. No factor had any significant association with the adjusted IGF-I and ghrelin levels at T3.

### 3.5. Effect of Fortification Protocols on Nutrient and Energy Intake

With the PTF protocol, the proportion of infants receiving protein, energy, and fat within the recommended range was high (88%, 96%, and 92%, respectively). The respective figures for the FF group were lower than in the PTF group, but the difference between the two groups was significant only for the protein intake. A high proportion of infants in the FF group received daily protein amounts higher than the recommended range. The estimated amount of lactose intake was higher than the upper recommended levels in 60% and 80% of the infants in the FF and PTF groups, respectively (Table S1).

## 4. Discussion

The risk of cardiovascular and metabolic disease and its relationship to the early nutrition and growth of preterm infants has gained considerable attention [3]. The “early protein” hypothesis suggests a programming effect of protein intake during the early postnatal life on neuro-endocrine and metabolic mechanisms of term infants [13]. Our plan was to investigate if the time-interval between the birth of a premature infant and the term equivalent age represents an additional “window of opportunity” by studying the relevant hormone changes following carefully planned nutrition intervention based on OMM. In the current study, the modified fortification protocol targeting the recommended daily protein intake (PTF) after adjustment to OMM protein content, gestational age and amount of daily milk intake resulted in significantly lower plasma IGF-I and higher ghrelin at the end of the intervention period compared to the FF, using the same multicomponent HMF formulation. IGF-1 positively correlated to protein intake and changes in z-score of weight, length, and BMI during the study period while ghrelin negatively correlated to protein and energy intake and changes in the z-score of BMI. Multivariate regression analysis revealed that the allocation group and protein intake were significant independent predictors of the adjusted IGF-I whereas the allocation group and energy were independent predictors of ghrelin levels at the end of intervention period. There were no significant differences in hormone levels at term equivalent age between the two groups.

Implementation of the modified, partly individualized, OMM fortification protocol achieved the recommended daily protein intake, while the fixed fortification protocol resulted in significantly higher daily protein intake using the same multicomponent HMF formulation. Moreover, the daily energy and fat intake was within the recommended range in a higher proportion of the neonates in the PTF group.

The “early protein intake” theory suggests that high protein intake during early life promotes growth in infants by stimulating IGF-I production through the inducing effect of branched-chain amino acids on insulin and IGF-I secretion [14,36,37,38]. Several studies in term infants, including two systematic reviews, showed that formula-fed infants have higher plasma IGF-I levels compared to the human milk-fed ones, which was attributed mainly to the higher protein content in infant formulas [12,13,14,39,40]. Studies in preterm infants showed that protein and/or energy intake have either a significant positive association [28] or no association [41,42] with IGF-I levels. The two fortification protocols used in the current study resulted in significantly different plasma IGF-I levels between the two study groups at the end of intervention period. The difference in IGF-I levels can be attributed to the different protein intake, which was found to be a significant independent predictor of the unadjusted and adjusted IGF-I values at the end of the intervention. In contrast, energy intake did not correlate with the unadjusted and adjusted IGF-I at any time-point. The later finding is in agreement with previous results in preterm infants, which did not demonstrate any significant association between IGF-I and energy intake during the first eight postnatal weeks [41], during the growth retardation phase [28], and up to six months of age [42].

In adults, plasma ghrelin is closely associated with food intake with levels rising during fasting and decreasing postprandially. For this reason, ghrelin fasting levels were obtained in all infants. In preterm infants, a study showed a significant positive association of energy intake on the second day of life with the respective ghrelin levels but no association on the third and sixth month after birth [43]. In contrast, another study did not demonstrate any significant effect of food intake on plasma ghrelin on the fourth day of life [44]. Results of the current study showed that the allocation group and energy intake were significant independent predictors of ghrelin at the end of intervention. However, the significant association between energy intake and ghrelin was eliminated after adjustment for the group allocation, indicating that this parameter, which reflects the difference in the total nutrient and energy intake, has a higher impact on ghrelin levels than the energy alone.

Several studies in preterm infants showed that IGF-I is associated positively with growth at various time points during the first 36 month of life [22,28,42,45,46,47]. Our findings support a positive effect of IGF-I on growth, as indicated by the positive correlation of IGF-I at either the end of intervention or at term equivalent age with changes in the z-scores of weight, length, and BMI during the study period. A positive correlation of IFG-I with weight and BMI z-scores was also reported in healthy term infants fed either breast milk or formula at the age of five months [46] as well as in preterm infants during the first eight weeks of life and at the age of nine and 36 months [22,28,47].

Regarding the association between ghrelin and growth parameters, increased levels have been reported in small for gestational age neonates [48], while most previous studies could not find any significant association in appropriate for gestational age infants [15,22,43]. However, a study in term and preterm neonates showed an inverse association between ghrelin and growth parameters, which however was limited to term neonates [24]. In the preterm population included in our study, ghrelin levels were not associated with the changes in z-scores of weight, length, and HC, but were inversely associated with the changes in z-score of BMI during intervention.

Regarding the effects of the fortification protocol on growth, we did not find any significant difference between the two groups up to the 12th month CA. Our findings are in agreement with previous studies in preterm infants fed fortified human milk with variable protein content [49,50,51]. However, a higher rate of weight gain in preterm infants receiving increased amounts of protein was reported by other authors [25,52,53,54,55,56]. Two meta-analyses exploring the effect of high protein or multi-component fortification of human milk, respectively, concluded that the protein supplemented human milk was associated with increased short-term growth in preterm infants, while the benefit of using multi-component supplementation was a slightly higher rate of weight gain during hospitalization [57,58].

Several FF protocols, with protein supplementation ranging between 0.8 g and 1.5 g per 100 mL of human milk, have been utilized in previous studies. However, the FF protocols do not take into consideration either the OMM nutrient content, which varies widely [5,59], or the daily amount of milk intake. The daily amount of milk intake by preterm infants ranges between 140 mL/kg/day and 200 mL/kg/day, depending on the estimated infant’s nutritional requirements according to the daily/weekly weight gain [6]. Consequently, protein intake following FF of human milk may range between being inadequate [60,61] to even being excessive [62]. In our study, the high daily volumes of OMM prescribed by the attending neonatologist (up to 180 mL per kg per day), combined with the lack of adjustment in the amount of fortifier in the FF group according to the volume of milk intake, may have attributed to the higher protein intake by this group. Our findings are in line with studies by de Halleux and Rigo, who found that, compared to the individualized fortification group, the FF group received higher protein amounts, which were greater than the recommended levels in about 40% of the preterm infants [62]. In contrast, other authors reported a lower protein intake by the FF group, with only 64% of them attaining the lower recommended protein levels [63,64]. The conflicting results could be attributed to the use of different methodologies. Specifically, in most studies, a proportion or all included infants were fed exclusively or partly banked donor milk [50,51,52,53,54,55], which contains lower protein than the preterm OMM [4,65], thereby requiring a higher fortification level. In other studies, a protein content of about 0.8–1.4 g/dL in human milk was assumed [49,50,51,54] or the protein intake was adjusted to blood urea levels [50,51,54,55].

Targeting the daily intake of an individual nutrient by using a multi-component HMF is challenging because the intake of other nutrients may be unpredictable. Therefore, we further analyzed the effect of the two fortification protocols on the intakes of all macronutrients. We found that the protein-targeting fortification protocol resulted in energy and fat intake within the recommended range in a higher proportion of infants compared to the FF protocol. Beyond any doubt individualized fortification of human or maternal milk is the preferred mode of feeding for preterm infants. However, when only multi-component HMFs are available, then a fortification protocol targeting the recommended protein intake can be an acceptable alternative to using multiple human milk supplements.

Changes in IGF-I and ghrelin levels during intrauterine life and early infancy have been implicated as potential factors predisposing to long-term metabolic and cardiovascular disturbances [66]. Our results suggest a transient effect of nutrient intake on IGF-I and ghrelin levels. However, in theory, even temporary changes of these hormones during vulnerable periods of life, such as intrauterine and early postnatal period, especially in the fast-developing preterm infants, may exert a programming effect on the neuroendocrine and metabolic pathways which could lead to long-term metabolic disturbances [9,37]. In this context, the results of the current study raise concerns about the potential long-term metabolic effects of modifications of mother’s milk fortification. Of note, the different IGF-I and ghrelin levels found in the two intervention groups should be interpreted with caution, because safety levels of these two hormones, i.e., levels not inducing metabolic/endocrine dysregulation, have not yet been established in preterm infants.

The main limitation of this study is the weekly assessment of OMM composition. Ideally, analysis of human milk should be performed at least twice a week [67]. However, for practical reasons, especially because mothers considered that expressing breast milk for this reason twice a week was very cumbersome, we opted for weekly analysis of OMM likewise several previous authors did [26,27,28]. Further research may clarify the potential clinical impact of the once and twice a week analysis of maternal milk. Another limitation is the fact that the mid-infrared milk analyzers cannot discriminate lactose from other non-digestible oligosaccharides. Previous studies reported a wide range of human milk oligosaccharide concentrations (12% to 30%), depending on gestational age, duration of breast feeding, ethnicity, maternal BMI, and other factors [29,32,33,34,35]. For this reason, we calculated lactose concentration, referred to as estimated lactose, and the lactose-derived energy of OMM assuming that oligosaccharides constitute 20% of total carbohydrates in human milk. The main strength of our study derives from the fact that it is the first to evaluate the effect of different HMF protocols on early IGF-I and ghrelin levels. Moreover, the single center design ensures that treatment and evaluation of all infants was uniformly based on the same department protocols.

## 5. Conclusions

Results of the present study showed that implementation of different OMM fortification protocols providing different amounts of protein is associated with changes in early IGF-I and ghrelin levels, which may have a programming effect on the neuroendocrine and metabolic mechanisms of preterm infants. These data suggests that the time interval between birth and 40th postconceptional week does represent a window of opportunity during which nutrition intervention carefully planned has the potential to influence the metabolism-related hormones. Although the influence on the relevant hormones seems temporary, the long-term effect on growth remains to be elucidated.

## Figures and Tables

**Table 1:** Clinical data of the two groups.

	FF Group	PTF Group	p *	Odds Ratio (5–95% CI)
*N*	23	25		
Gestational age (wks)	28.6 ± 1.9	28.3 ± 1.6	0.433	N.A.
Birth weight (g)	1156 ± 208	1149 ± 185	0.794	N.A.
Birth weight *z*-score	−0.14 ± 0.68	−0.07 ± 0.69	0.260	N.A.
Birth weight < 1200 g, *n* (%)	15 (65.2)	13 (52.0)	0.394	1.731 (0.541–5.54)
Ponderal index	22.7 ± 2.2	23.6 ± 3.0	0.421	N.A.
Cesarean section, *n* (%)	19 (83)	17 (68)	0.324	0.447 (0.114–1.76)
Male sex, *n* (%)	8 (35)	11 (44)	0.566	0.68 (0.211–2.18)
Prenatal steroids, *n* (%)	17 (74)	20 (77)	0.736	0.708 (0.183–7.74)
Respiratory distress syndrome, *n* (%)	21 (91)	24 (96)	0.601	0.438 (0.037–5.18)
Intraventricular hemorrhage I-II, *n* (%)	1 (4.3)	3 (1205)	0.610	0.333 (0.032–3.46)
Bronchopulmonary dysplasia (mild), *n* (%)	7 (30)	6 (24)	0.748	1.385 (0.886–4.95)
Patent ductus arteriosus, *n* (%)	5 (22)	5 (20)	1.0	1.111 (0.276–2.04)
ROP requiring intervention, *n* (%)	1 (4)	0 (0)	1.0	N.A.
Postnatal age at T1 (d)	10.1 ± 3.6	11.4 ± 5.7	0.574	N.A.
Postnatal age at T2 (d)	40.8 ± 11.9	41.9 ± 12.0	0.794	N.A.
Postnatal age at T3 (d)	77.5 ± 13.1	80.4 ± 11.6	0.451	N.A.
Days on intervention	30.7 ± 10.9	30.5 ± 11.8	0.996	N.A.
Days in Hospital	68.9 ± 24.0	70.6 ± 25.7	0.947	N.A.

FF, fixed fortification; PTF, protein-targeting fortification; T1, start of intervention; T2, end of intervention; T3, 40th postconceptional week; d, days; wks, weeks; ROP, retinopathy of prematurity; N.A, not applicable. Continuous variables are expressed as means ±SD. * Mann–Whitney test, or Fisher’s exact test, as appropriate.

**Table 2:** Mean daily macronutrient and energy intake during the intervention period and changes in *z*-scores of growth parameters during the study period.

	FF Group (*n* = 23)	PTF Group (*n* = 25)	*p* (Mann-Whitney)
Protein (g/kg/d)	4.2 ± 0.4	4.0 ± 0.3	0.028
Energy (kcal/kg/d) *	130 ± 9	124 ± 9	0.060
Protein/energy ratio *	3.2 ± 0.3	3.3 ± 0.3	0.931
Fat (g/kg/d)	6.1 ± 1.0	6.0 ± 0.7	0.980
Carbohydrates (g/kg/d)	16.1 ± 0.9	14.8 ± 1.0	<0.001
Estimated lactose (g/kg/d) *	14.2 ± 0.7	12.8 ± 1.1	<0.001
Milk (mL/kg/d)	160 ± 8	162 ± 10	0.265
Mean HMF (g/kg/d)	7.4 ± 1.4	5.4 ± 1.2	<0.001
ΔWeight z-score T1 to T2	−0.02 ± 0.58	−0.13 ± 0.44	0.172
ΔWeight z-score T1 to T3	0.28 ± 0.85	0.19 ± 0.84	0.483
ΔLength z-score T1 to T2	−0.33 ± 0.59	−0.50 ± 0.58	0.521
ΔLength z-score T1 to T3	−0.45 ± 0.67	−0.42 ± 0.97	0.746
ΔHC z-score T1 to T2	0.30 ± 0.72	0.24 ± 0.78	0.786
ΔHC z-score T1 to T3	0.63 ± 0.98	0.68 ± 0.84	0.980
ΔBMI z-score T1 to T2	0.85 ± 1.05	0.29 ± 1.75	0.343
ΔBMI z-score T1 to T3	1.01 ± 1.14	0.57 ± 2.13	0.516

* Lactose and energy intake were calculated assuming a 20% of total carbohydrates as non-metabolized oligosaccharides. FF, fixed fortification; PTF, protein-targeting fortification; HMF, human milk fortifier; HC, head circumference; Δ, difference; d, days; Values are expressed as means ±SD.

**Table 3:** Plasma levels [median (min-max)] of IGF-I and ghrelin at T2 and T3 in the two intervention groups.

	FF Group (*n* = 23)	PTF Group (*n* = 25)	*p* (Mann–Whitney U)
IGF-I T2 (ng/mL)	31.0 (20–93)	19.2 (10.9–89.1)	0.025
IGF-I T3 (ng/mL)	36.1 (10.6–112.3)	46.2 (10.7–86.6)	0.458
Ghrelin T2 (pg/mL)	396 (163–674)	483 (232–966)	0.045
Ghrelin T3 (pg/mL)	364 (163–1140)	411 (187–1210)	0.248

**Table 4:** Correlations of IGF I and ghrelin at T2 and T3 with nutrient intake, energy intake, and changes in z-scores of growth parameters during intervention (T1 to T2) and up to the term equivalent age (T1 to T3) using the Spearman’s correlation coefficient.

	IGF-I T2	Ghrelin T2	IGF T3	Ghrelin T3
	*p* (r)	*p* (r)	*p* (r)	*p* (r)
Mean daily protein intake	0.039 (0.299)	0.873 (−0.024)	0.273 (0.161)	0.018 (−0.341)
Mean daily energy intake	0.277 (0.160)	0.031 (−0.312)	0.675 (0.062)	0.033 (−0.309)
Mean protein/energy ratio	0.060 (0.273)	0.176 (0.199)	0.420 (0.119)	0.996 (−0.001)
Δ *z*-score of weight T1–T2	0.604 (0.077)	0.052 (−0.282)	0.043 (0.298)	0.112 (−0.232)
Δ *z*-score of weight T1–T3	0.659 (−0.065)	0.657 (−0.066)	0.403 (0.121)	0.992 (−0.002)
Δ *z*-score of length T1–T2	0.001 (0.446)	0.751 (0.047)	0.714 (0.054)	0.497 (0.100)
Δ *z*-score of length T1–T3	0.044 (0.292)	0.647 (0.068)	0.068 (−0.265)	0.412 (0.121)
Δ *z*-score of HC T1–T2	0.169 (0.234)	0.093 (−0.245)	0.563 (0.086)	0.621 (0.073)
Δ *z*-score HC of T1–T3	0.910 (−0.017)	0.337 (−0.141)	0.690 (−0.059)	0.294 (0.155)
Δ *z*-score of BMI T1–T2	0.095 (−0.245)	0.002 (−0.432)	0.185 (0.194)	0.037 (−0.302)
Δ *z*-score of BMI T1–T3	0.357(−0.136)	0.182 (−0.196)	0.040 (0.297)	0.181 (−0.196)

HC, head circumference; Δ, changes.

**Table 5:** Multiple regression analysis (generalized linear model) with dependent variables the natural logarithms of IGF-I and ghrelin levels at T2 and T3, in separate models.

Independent Variables	Dependent Variables							
	B	95% Wald CI (Lower/Upper)	*p*	Exp (B)	B	95% Wald CI (Lower/Upper)	*p*	Exp (B)
	IGF I LOG at T2				IGF I LOG at T3			
**MODEL 1**								
Intervention group	0.329	0.001/0.658	0.049	1.390	0.051	−0.419/0.521	0.832	1.052
Protein intake	0.506	0.010/1.001	0.045	1.658	0.250	−0.460/0.960	0.490	1.284
Energy intake	−0.009	−0.197/0.011	0.360	0.991	−0.004	−0.033/0.024	0.760	0.996
**MODEL 2**								
Protein intake	0.572	0.062/1.083	0.028	1.772	0.260	−0.444/0.964	0.461	1.297
Energy intake	−0.005	−0.025/0.015	0.650	0.995	−0.004	−0.031/0.024	0.792	0.996
	**GHRELIN LOG at T2**				**GHRELIN LOG at T3**			
**MODEL 1**								
Intervention group	−0.302	−0.567/−0.037	0.025	0.739	−0.023	−0.329/0.283	0.883	0.977
Protein intake	0.162	−0.238/0.562	0.425	1.176	−0.358	−0.820/−0.104	0.129	0.699
Energy intake	−0.014	−0.030/0.002	0.096	0.986	−0.012	−0.030/0.007	0.208	0.988
**MODEL 2**								
Protein intake	0.101	−0.315/0.518	0.633	1.107	−0.363	−0.821/−0.095	0.121	0.696
Energy intake	−0.018	−0.034/0.001	**0.033**	0.982	−0.012	−0.030/0.006	0.184	0.988

CI, confidence intervals.

## Supplementary Materials

The following are available online at https://www.mdpi.com/2072-6643/11/12/3056/s1, Supplementary Method text: Breast milk collection protocol and analysis; Table S1: Numbers (%) of infants receiving daily nutrient and energy intake within or out of the recommended range.
