# Supplementary material for: Modifications of Own Mothers’ Milk Fortification Protocol Affect Early Plasma IGF-I and Ghrelin Levels in Preterm Infants. A Randomized Clinical Trial
Source: Nutrients. 2019 Dec 14;11(12):3056. doi: 10.3390/nu11123056 (PMC6950485; doi:10.3390/nu11123056)
Supplement: Supplementary file 1 [file nutrients-11-03056-s001.pdf]

Supplementary Material

Breast milk collection protocol and analysis

Soon after delivery, all mothers eligible for enrollment received written and verbal instructions on breast milk collection and proper procedures of breast emptying using an electrical pump, hygiene rules, milk extraction, proper storage, and transferring to the hospital. The parents' compliance to the instructions was checked regularly. Mothers were advised to pump the milk every three hours for at least 15 minutes on each breast, starting from the first day after delivery to ensure the extraction of sufficient amounts of milk for both feeding the infant and sampling for the analysis. Additionally, the mothers were encouraged to activate the mammary gland (breast massage) and completely empty it during pumping. Each mother was provided with milk collection containers and equipment for transporting the milk, to prevent contamination and facilitate the process. Analysis of OMM was performed in both groups starting on the day prior to T1 and was repeated at weekly intervals, on the same day every week, as performed by previous investigators [S1-S4]. Attempts to obtain milk samples twice a week were unsuccessful due to maternal dissatisfaction. For analysis of OMM, three samples (5mL each) were obtained from three different meals (morning, afternoon, evening) of the day. The sample was obtained after manual homogenization of the whole meal volume. Then, samples were refrigerated at 4°C until next morning when the three samples of the day were transported to the laboratory. Prior to analysis, the three samples were warmed to 40°C and mixed in a plastic container by gentle inversion for about 3 minutes to ensure homogeneity and prevent fat globules from sticking to the bottle wall. Analysis was performed by applying mid-infrared spectrometry, using the Milkoscan TM Minor (FOSS Analytical A/S, Hillerod, Denmark). The analyzer was calibrated, according to ISO 9622-IDF (International Dairy Federation) 141-2013, against chemical reference methods for the quantification of macronutrient content in breast milk. For more accurate and precise measurements, calibration was performed using breast milk samples that represented the broad range of variation in macronutrient content in preterm

and term milk. Calibration of the analyzer and measurement of breast milk samples were performed according to the manufacturer's instructions. Measurements were performed in duplicate.

Table S1. Numbers (%) of infants receiving a daily nutrient and energy intake within or out of the recommended range [S5]

	Intake levels in relation to the recommended	Fixed fortification group	Protein-adjusted fortification group	P (Fisher's exact test)
Protein	Low	3 (13.0)	1 (4.0)	
	Within	7 (30.4)	22 (88.0)	<0.001
	High	13 (56.5)	2 (8.0)	
Energy	Low	0	1 (4.0)	
	Within	20 (87.0)	24 (96.0)	0.102
	High	3 (13.0)	0	
Estimated lactose	Low	0	0	
	Within	9 (39.1)	5 (20.0)	0.207
	High	14 (60.9)	20 (80.0)	
Fat	Low	0	0	
	Within	17 (73.9)	23 (92.0)	0.130
	High	6 (26.1)	2 (8.0)	

References

S1. Miller, J.; Makrides, M.; Gibson, R.A.; McPhee, A.J.; Stanford, T.E.; Morris, S.; Ryan, P.; Collins, C.T. Effect of increasing protein content of human milk fortifier on growth in preterm infants born at 31 wk gestation: a randomized controlled trial. *Am J Clin Nutr* **2012**, *95*, 648-655. doi: 10.3945/ajcn.111.026351.

S2. Zachariassen, G.; Fenger-Gron, J.; Hviid, M.V.; Halken, S. The content of macronutrients in milk from mothers of very preterm infants is highly variable. *Dan Med J* **2013**, *60*(6), A4631, PMID: 23743111.

S3. Bauer, J.; Gerss, J. Longitudinal analysis of macronutrients and minerals in human milk produced by mothers of preterm infants. *Clin Nutr* **2011**, *30*, 215-220. doi:10.1016/j.clnu.2010.08.003

S4. Hansen-Pupp, I.; Löfqvist, C.; Polberger, S.; Niklasson, A.; Fellman, V.; Hellström, A.; Lay, D. Influence of insulin-like growth factor I and nutrition during phases of postnatal growth in very preterm infants. *Pediatr Res* **2011**, *69*, 448-453. doi: 10.1203/PDR.0b013e3182115000.

S5. Agostoni, C.; Buonocore, G.; Carnielli, V.P.; de Curtis, M.; Darmaun, D.; Decsi, T.; Domellöf, M.; Embleton, N.D.; Fusch, C.; Genzel-Boroviczeny, O.; et al. ESPGHAN Committee on Nutrition. Enteral nutrient supply for preterm infants: commentary from the European Society of Paediatric Gastroenterology, Hepatology, and Nutrition Committee on Nutrition. *J Pediatr Gastroenterol Nutr* **2010**, *50*, 85-91. doi: 10.1097/MPG.0b013e3181adaee0
